# Features of the immunosuppressive tumor microenvironment in endometrial cancer based on molecular subtype

**DOI:** 10.3389/fonc.2023.1278863

**Published:** 2023-10-20

**Authors:** Chong Zhang, Ming Wang, Yumei Wu

**Affiliations:** ^1^ Departments of Obstetrics, Beijing You’an Hospital of Capital Medical University, Beijing, China; ^2^ Department of Gynecologic Oncology, Beijing Obstetrics and Gynecology Hospital, Capital Medical University, Beijing Maternal and Child Health Care Hospital, Beijing, China

**Keywords:** endometrial cancer, immunosuppressive tumor microenvironment, immunophenotype, molecular subtypes, immunotherapy

## Abstract

Endometrial cancer (EC) is one of the three most prevalent gynecological tumors affecting women and is the most prevalent gynecological malignancy in the developed world. Its incidence is rapidly increasing worldwide, mostly affecting postmenopausal women, whereas recently its prevalence has increased in younger people. EC is an immune gene disease and many studies have shown that the tumor-immunosuppressive microenvironment plays an important role in cancer progression. In recent years, findings regarding the immunosuppressive tumor microenvironment (ITME) of EC have included immune evasion mechanisms and immunotherapy, which are mostly immune checkpoint inhibitors (ICI) for EC. Recently studies on the ITME of different molecular types of EC have found that different molecular types may have different ITME. With the research on the immune microenvironment of EC, a new immunophenotype classification based on the immune microenvironment has been carried out in recent years. However, the impact of the ITME on EC remains unclear, and the immunophenotype of EC remains limited to the research stage. Our review describes recent findings regarding the ITME features of different EC molecular types. The advent of immunotherapy has brought hope for improved efficacy and prognosis in patients with advanced or recurrent EC. The efficacy and safety of ICIs combination therapy remains the focus of future research.

## 1 Introduction

Endometrial cancer (EC) is an immune gene disease, and many studies have suggested that the immunosuppressive tumor microenvironment (ITME) plays an important role in cancer progression (1–3). EC is commonly diagnosed at an early stage because abnormal bleeding is a common clinical symptom with a favorable prognosis (five-year overall survival: 71-80%) (4). For patients with advanced disease before symptoms appear, the prognosis is worse and they respond poorly to conventional therapies (5). Therefore, in recent years, immunotherapy has attracted attention, and researchers have conducted studies on the ITME of EC. Since 2014, the Food and Drug Administration (FDA) has approved several immune checkpoint inhibitors (ICI) in clinical practice, mainly for patients with late-stage or recurrent cancers for whom routine treatment has failed. However, the relatively low response rate limits its application in clinical practice, and the efficacy of immunotherapy is unsatisfactory (6). Therefore, screening populations for therapeutic advantages when using ICIs is crucial. Classification methods for EC are constantly improving, gradually shifting from traditional clinical and pathological classifications to molecular classifications. Therefore, studying the ITME of different molecular subtypes is important. Recently studies on the ITME of different molecular types of EC have revealed that different molecular types may have different ITME (7, 8). However impact of the ITME on EC remains unclear. Our review describes recent findings regarding the ITME features of different EC molecular types.

## 2 Main body

### 2.1 Molecular types of EC

EC has been broadly divided into two groups based on histomorphology since 1983. Type I is endometrioid tumors, which are the most common subtype, and most of the tumors express the estrogen receptor (ER) and have a favorable prognosis. In contrast, type II is estrogen-independent and mainly represents a serous carcinoma with a poor outcome (9, 10). However, the classification based on tumor histopathology is subjective, has reproducibility challenges, and high-level EC cannot be reliably based on histological criteria. Misdiagnosis is possible due to the presence of mixed high-grade histological components. Biological information to enhance diagnosis is urgently required in this situation.

**Figure 1:**
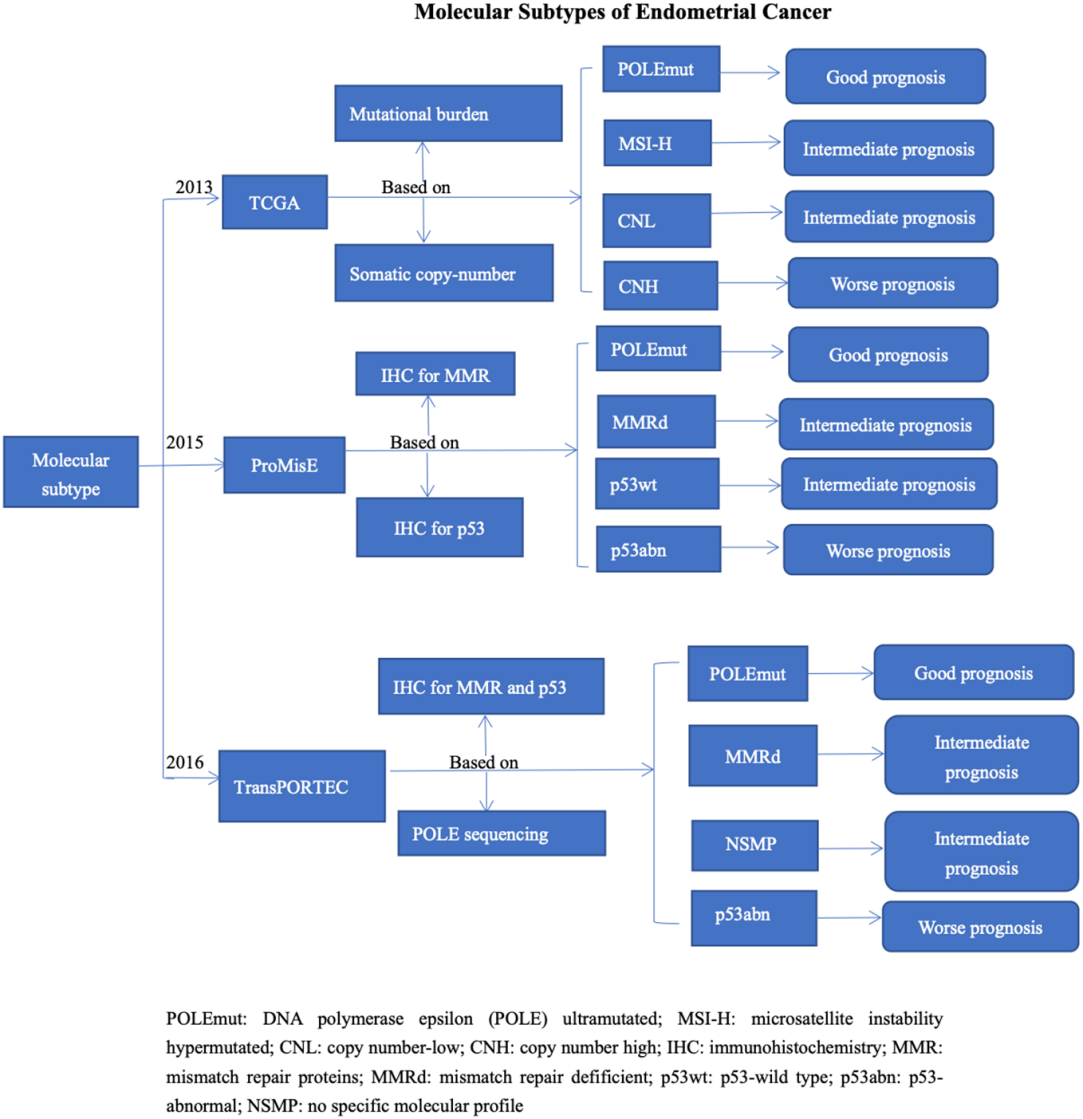
Molecular Subtypes of Endometrial Cancer.

To overcome these limitations, the Cancer Genome Atlas (TCGA) Research Network, based on mutational burden and somatic copy-number variations, revealed four molecular subtypes of EC with distinct prognoses: DNA polymerase epsilon (POLE) ultramutated (POLEmut) with an excellent prognosis, microsatellite instability hypermutated (MSI-H) with an intermediate prognosis, copy number high (CNH) with the worst prognosis, and copy number low (CNL) with an intermediate prognosis (11). However, the TCGA studies only include endometrioid and serous EC, and the additional cost of entire genome sequencing greatly limits its practical application. Subsequent studies have found cheaper and easier surrogates. To broaden the utility of TCGA classification, Talhouk proposed a practical molecular POLE mutation-based classification model, the Proactive Molecular Risk Classififier for Endometrial Cance (ProMisE) (12). In this study, immunohistochemistry (IHC) for mismatch repair (MMR) proteins and immunohistochemical staining for p53 were used instead of molecular assessments of mutational burden and copy number variations. Therefore, the ProMisE model classified four subgroups: mismatch repair deficient (MMRd), POLE-ultramutated (POLEmut), p53-wild type (p53wt), and p53-abnormal (p53abn) (13, 14). In 2016, Stelloo et al. validated a more pragmatic, cost-effective, and clinically applicable molecular classification system called the Translational Research in Post-Operative Radiation Therapy in EC (TransPORTEC) system. Therefore, EC can be subdivided into four subgroups: the POLE-mutated, MMRd, p53abn, and no specific molecular profile (NSMP) (15, 16). POLE-mutant ECs have a highly favorable prognosis and do not require adjuvant treatment. MMRd ECs have an intermediate prognosis. Numerous recent studies have focused on the immunotherapy for EC (17, 18). And reports have demonstrated these two groups may benefit from immunotherapy (19, 20). P53-mutant ECs are typically associated with more advanced stages, higher rates of lymphatic vascular space infiltration (LVSI), and diverse pathological types. Most P53-mutant ECs are serous adenocarcinomas and have a poor prognosis. The NSMP group was the most frequent, and its prognosis was uncertain (16), as shown in **Figure 1**.

In view of the important prognostic and therapeutic implications of the European Society of Gynecological Oncology (ESGO), European Society of Pathology (ESP), and European Society for Radiotherapy and Oncology (ESTRO) guidelines, new prognostic risk groups were defined of adopting the molecular classification for EC treatment in 2020 (21). ECs with POLE-mutant confined to the uterus are regarded as low-risk, whereas p53-abnormal ECs are regarded as “high-risk” in the presence of invasion (22).

### 2.2 Tumor immunosuppressive microenvironment in EC

The tumor microenvironment plays a crucial role in the occurrence and development of malignant tumors, and has a significant impact on the clinical outcomes of EC (23). Immune cells in a normal endometrium are vital for protection against external pathogens. The immune cells, mesenchymal cells, endothelial cells, inflammatory mediators, and extracellular matrix (ECM) molecules are components of the ITME. Infiltrating stromal and immune cells are the major components of the ITME and play a significant role in the biological behavior of cancer (24). Related studies have shown that many immune cells and cytokines are found in EC tissues and can stimulate an endogenous anti-tumor immune response. Therefore, these patients may benefit from immunotherapy (25–27).

Tumor-infiltrating immune cells can exert an anti-tumor killing effect through a specific cellular immune-mediated immune response. Some subsets can control the growth and progression of tumors, whereas others can promote immune suppression and help tumor cells escape the immune system. Finally, the body reaches a balance between the tumor immune response and tumor immune escape (28, 29). Tumor-infiltrating immune cells include T cells, tumor-associated macrophages (TAMs), natural killer (NK) cells, dendritic cells (DCs), and B cells. (**Table 1**) T cells can be divided into two subgroups: CD4^+^ and CD8^+^. In terms of immune function, CD4^+^ T cells are mainly regulatory T cells (Tregs), which assist humoral and cellular immunity by secreting various lymphokines. CD8^+^ T cells mainly include inhibitory and cytotoxic T cells. CD8^+^ inhibitory T cells can inhibit humoral immunity. CD8^+^ cytotoxic T lymphocytes (CTLs) can lead to the apoptosis of target cells by releasing perforin, granzyme killer cells, or through the Fas/FasL pathway (30, 31). Cytokines are immune checkpoint molecules and inflammatory factors. The immune checkpoint molecules include members of the B7 family, human leukocyte antigen 1(HLA 1) and lymphocyte activation gene-3 (LAG-3) are shown in. **Table 2** (61–64). Inflammatory factors includes interleukin−6 (IL−6), IL-11, tumor necrosis factor a (TNF-α), Cox-2 and interferon.

**Table 1:** Tumor-infiltrating immune cells in immunosuppressive tumor microenvironment.

Regulatory function	Immune cell	Role	Clinical studies and findings
Down-regulation	CD8+CTL	Induce the apoptosis of target cells (30, 31).	The number of CD8^+^ CTLs is lower in the endometrium of EC patients than in the normal endometrium (32). CD8^+^ CTLs generally indicate a good prognosis and a lower T-cell density has been found in advanced-stage ECs (26). CD8+ CTLs can be used as an independent predictor of OS (33).
	DC	Identify tumor antigen presentation signals, induce the generation of CD8^+^ CTLs, and secrete cytokines (34).	The reduction of DC activity was associated with EC progression (35). There are morphological differences in the DCs of the endometrium between patients with EC and healthy humans, and the endometrium in patients had lower levels of CD80, CD86, and CD40, which are expressed on DCs (36).
	NK	Be activated by various cytokines and have cell-killing functions (37).	Mean values of NK cell activity were significantly lower in patients with stages I and II of EC when compared with healthy controls (38). NK cell activity in stage I EC patients was negatively associated with nuclear grading, myometrial invasion, and immunoreactivity of proliferating cell nuclear antigen (39).
Up-regulation	TAM	Express many anti-inflammatory factors, such as IL-10 and TGF-β (40).	Most studies reported a positive correlation between the expression of TAMs and advanced stage, RFS and OS in patients with EC (41–43).
	Treg	Mediate the immune escape of tumor cells and promote tumor metastasis and progression (44).	Tregs expression was significantly increased in the tumor groups of all grades compared to the normal endometrial group (44). The upregulation of CD4 expression in T cells in the ITME was positively associated with high cancer grade, cancer stage, and myometrium invasion (45).

CD8^+^CTL, CD8^+^ T lymphocytes; DC, dendritic cells; NK, Natural killer cells; TAM, tumor associated macrophages; Treg, regulatory T cells; OS, overall survival; RFS, recurrence-free survival.

**Table 2:** Immune checkpoint molecules in immunosuppressive tumor microenvironment.

Regulatory function	Immune checkpoint molecules	Mechanism	Clinical studies and findings
Up-regulation	PD-1/PD-L1	PD-1 combined with PD-L1 can inhibit the proliferation and differentiation of T cells (46).	The expression levels of PD-1 and PD-L1 are related to a poor prognosis (47). The expression levels of PD-1 and PD-L1 are related to the degree of T lymphocyte infiltration in POLEmut and MSI-H EC (48, 49). Both POLEmut and MSI-H type EC patients are more sensitive to immunotherapy based on PD-1/PD-L1 inhibitors (48, 49).
	CTLA-4	Inhibit CTL activation by preventing the binding of B7 ligand to CD28 (50).	The combined blockade of CTLA-4 and PD-1 amplifies anti-tumor T cell responses and provides synergistic activity (51).
	B7-H3	A negative regulatory factor for T-cell activation and may protect tumor cells from immune system surveillance (52).	B7-H3 is expressed in most EC, and its high expression is closely associated with high-risk tumors (52). The expression of B7−H3 in EC cells positively correlated with the frequency of CD8^+^ TILs and the overexpression of B7−H3 in EC cells was associated with short OS time (52).
	B7-H4	Inhibit T lymphocyte proliferation and cytokine secretion (53).	B7−H4 was upregulated in malignant EC and was more common in advanced subgroups (54). The expression of B7−H4 was an independent factor of EC grade, histological type, and infiltrating−immune cell type (55).
	HLA	Present immunogenic peptides to CTL (56).	HLA class I molecules were downregulated on the surface of the EC (56). HLA was associated with high grade of EC (57). The HLA level in patients with early stages of EC was high (58).
	LAG-3	Iinhibit immune cell proliferation and cytokine release (59).	LAG-3 expression in immune cells was more common in high-grade, high-intermediate risk, high-risk, and advanced/metastatic subgroups and was relevant to lymphovascular space invasion (60). LAG-3 expression was more prevalent in POLEmut and MMRd EC than in p53abn and p53wt EC in tumor cells Positive LAG-3 expression may be a predictor of improved RFS (60).

PD−1, programmed cell death 1; PD−L1, programmed cell death 1 ligand 1; CTLA-4, cytotoxic T-lymphocyte-associated protein 4; B7-H3, B7 homolog 3; TILs, tumor-infiltrating lymphocytes; B7-H4, B7 homolog 4; HLA, human leukocyte antigen; LAG-3, lymphocyte activation gene-3; EC, endometrial cancer; POLEmut, DNA polymerase epsilon ultramutated; MMRd, mismatch repair deficient; MSI-H, microsatellite instability hypermutated; RFS, recurrence-free survival.

### 2.3 Tumor-infiltrating immune cells

#### 2.3.1 CD8^+^ CTLs

CD8^+^ CTLs can induce the apoptosis of target cells (30, 31). CD8^+^ CTLs have been isolated from the peripheral blood or tumor tissues of patients with a variety of cancers, such as melanoma and lung cancer. This may have potential value in the early diagnosis of tumors (65). In terms of EC, reports have demonstrated that the number of CD8^+^ CTLs is lower in the endometrium of EC patients than in the normal endometrium (32). Studies have found that tumors with fewer somatic mutations produced lower levels of immunogenic antigens (66). Therefore, CD8^+^ CTLs generally indicate a good prognosis and a lower T-cell density has been found in advanced-stage ECs (26). Furthermore, Suemori (33) analyzed 123 cases of EC tissues using immunohistochemistry (IHC) and found that CD8^+^ CTLs can be used as an independent predictors of the overall survival rate (OS) of EC. Dai et al. (7) conducted a retrospective study of 26 patients with EC and observed relatively high percentages of CD8^+^ CTLs in TMB-high EC samples, which were considered to have a good survival outcome. Kondratiev et al. (67) studied 90 patients with EC and found that an increased number of CD8^+^ CTLs in the epithelial cells at the tumor-invasive border was a favorable prognostic factor for EC patients. Compared to patients with a lower number of CD8^+^ CTLs in the epithelium at the infiltrating boundary of tumors, patients with a higher number of CD8^+^ CTLs showed an improvement in overall survival (OS) time. The study showed that tumor stage, tumor grade, vascular invasion, and the number of CD8^+^ CTLs in ITME were independent predictors of OS (67). All the above studies illustrate the value of CD8^+^ CTLs in the prognostic evaluation of EC. Dynamic monitoring of changes in CD8^+^ CTLs in patients EC has great clinical reference value for judging prognosis, guiding treatment, and follow-up management (26, 33, 68).

#### 2.3.2 TAMs

TAMs are important factors that affect the tumor microenvironment and can lead to drug resistance by promoting tumor invasion, metastasis, and blood vessel formation (69). TAMs are a diverse subset of tumor-infiltrating immune cells derived from monocytes that exhibit traditional cytotoxicity and phagocytic properties (70). Macrophages can be induced to form two major phenotypes: classically activated macrophages (M1) and selectively activated macrophages (M2). M1 type macrophages express a range of pro-inflammatory cytokines, chemokines and effector molecules, such as IL-12, IL-23, TNF-α and MHC I/II. In contrast, the M2 type macrophages express many anti-inflammatory factors, such as IL-10 and TGF-β. TAMs are classified as M2 type macrophages (40). Wang et al. (68) found that TAMs are the most abundant TME-infiltrating cells, followed by CD8^+^ CTLs. Currently, whether measuring TAM density in EC has clinical or prognostic significance is controversial. Hannah (26) reported no correlation between TAM density and cancer progression. Kübler et al. (41) reported a positive correlation between the expression of TAMs and advanced stage, recurrence-free survival (RFS), and OS in patients with EC. Dun et al. (42) reported that compared with normal endometrial cells, CD68^+^ macrophages were more abundant in the epithelial and stromal cells of type I and type II EC. Furthermore, Soeda et al. (43) compared patients with low CD68^+^ TAM density with EC patients who had higher CD68^+^ macrophage counts in ITME and found that they had worse progression−free survival (PFS) and OS time.

#### 2.3.3 Tregs

Tregs are immunosuppressive regulatory cells that are incapable of preventing excessive immune responses in a various tumor tissues. Studies have shown that Tregs mediate the immune escape of tumor cells and promote tumor metastasis and progression. A balance exists between the numbers of Tregs and CD8^+^ CTLs, which is crucial for the establishment of an effective immune monitoring system. The increase in the Tregs/CD8^+^ CTLs ratio indicated a poor anti-tumor effect. In addition, Julie et al. (44) reported that Tregs expression was significantly increased in the tumor groups of all grades compared to the normal endometrial group. In a study involving 57 patients with stage I−IV EC, Chang et al. (45) found that the number of CD4^+^ T cells was higher in the infiltrating lymphocytes of tumors than in the peripheral blood lymphocytes. Furthermore, they showed that the upregulation of CD4 expression in T cells in the ITME was positively associated with high cancer grade, cancer stage, and myometrium invasion (45). Another study reported that the infiltration of CD4^+^ Tregs into the ITME is relevant to the prognosis of patients with EC (71). All evidence suggests that Tregs are indicators of poor prognosis in patients with EC (72).

#### 2.3.4 DCs

DCs are the most powerful antigen presenting cells in the human body. They can identify tumor antigen presentation signals, induce the generation of CD8^+^ CTLs, and secrete cytokines during anti-tumor immune processes. Tumor cells cannot be recognized or presented by DCs owing to their low antigenicity. Therefore, CD8^+^ CTLs cannot be activated, leading to tumor cell escape and continued tumor growth. Effective identification of tumor cells is the first step in immunotherapy (34). Li et al. reported that DCs are significantly associated with survival patients with EC (73). Chen et al. (35) suggested that a reduction in DC activity is associated with EC progression. Studies have also shown that there are morphological differences in the DCs of the endometrium between patients with EC and healthy humans, and the endometrium in patients had lower levels of CD80, CD86, and CD40, which are expressed on DCs (36). This evidence reflected that the function of tumor−infiltrating DCs in tumor microenvironment was downregulated which affected antigen presentation, and promoted immune escape of tumor. In 2010, the FDA approved the peptide DC vaccine Sipuleucel-T for the treatment of refractory prostate cancer (74). Currently many DC vaccines are in different stages of clinical trials. Studies on DC vaccine applications in EC treatment have found that activated DC have an obvious killing effect on EC cells (75).

#### 2.3.5 NK cells

NK cells are a class of innate immune cells with cytotoxicity similar to that of CD8^+^ CTLs and both of are anti-tumor effector cells. The activation and cytotoxicity of NK cells depend on the balance between the inhibitory and activation signals. NK cells are activated by various cytokines and have cell-killing functions (37). Although NK cells kill tumor cells, if this balance is disrupted, they can promote tumor growth and tissue infiltration during the immune escape of EC tumors. Human leukocytes antigens (HLA), co-inhibitory molecules, and inhibitory cytokines can also activate NK cells, but the inhibitory signals provided by them impairs the cytotoxic function of NK cells. The abundance of immunosuppressive molecules present in the EC tumor microenvironment is involved in EC progression by affecting NK cell function (76). Garzetti et al. (38) reported that the mean values of NK cell activity were significantly lower in patients with stages I and II of EC when compared with healthy controls. The authors suggested that a decrease in NK cell activity was associated with the depth of myometrial invasion (38). In the following research, Garzetti et al. (39) made further studies about the activity of NK cells and found that NK cell activity in stage I EC patients was negatively associated with nuclear grading, myometrial invasion, and immunoreactivity of proliferating cell nuclear antigen.

### 2.4 Immune checkpoint molecules

#### 2.4.1 B7 family

The B7 family of immune checkpoint molecules is divided into three subgroups. Group I comprises B7-1, B7-2, CD28, Cytotoxic T-lymphocyte-associated protein 4 (CTLA-4), and B7H. Group II comprises programmed cell death 1 (PD−1)/programmed cell death 1 ligand 1(PD−L1). Group III comprises B7 homolog 3 (B7−H3), B7 homolog 4(B7-H4), HERV−H LTR−associating 2, and transmembrane and immunoglobulin domain-containing protein 2. Members of the B7 family play a critical role in the immune response and immune escape (77).

##### 2.4.1.1 PD-1/PD-L1

PD-1 is an important inhibitory co-stimulatory molecule and is expressed on the surface of activated T cells. Combined with its ligand, PD-L1 can inhibit the proliferation and differentiation of T cells, cause T cells to be in an inhibitory state, reduce their lethality to tumor cells, and lead to the immune escape of tumor cells. PD-1/PD-L1 inhibitors can relieve the negative immune regulatory effect of PD 1/PD L1 on T cells, promote effector T cell-specific recognition, and kill tumor cells (46). To escape the immune system, EC cells can stimulate immune checkpoints to activate negative feedback mechanisms and establish a local ITME. PD-1/PD -L1 inhibitors cause an excessive immune response and tissue damage. Liu et al. (78) found that the positive expression rates of PD-L1 in primary, recurrent, and metastatic endometrial cancers were 83%, 68%, and 100%, respectively by immunohistochemistry. Previous studies showed that PD-1 and PD-L1 are expressed in most EC tissues. The higher the level of PD-L1 expression, the worse is the differentiation of the tissue. Vanderstraeten et al. (79) found that the expression rate of PD-L1 in primary and metastatic EC tumor cells were 83% and 100%, respectively. In type II EC, the expression rates of PD-1 in the tumor tissue and the tumor microenvironment were 42% and 53%, respectively. And the expression rate of PD-L1 in the tumor tissue and tumor microenvironment were 15% and 28%, respectively. All the evidence indicates that the expression levels of PD-1 and PD-L1 are associated with a poor prognosis (47). Eggink (48) and Howitt (49) confirmed that the expression levels of PD-1 and PD-L1 are related to the degree of T lymphocyte infiltration in POLEmut and MSI-H EC. The higher the number of T lymphocytes, the stronger the local immune response and the better the prognosis. Therefore, patients with both POLEmut and MSI-H-type EC are more sensitive to immunotherapies based on PD-1/PD-L1 inhibitors.

##### 2.4.1.2 CTLA-4

CTLA-4, also called CD152, is regarded as a negative immune regulator and is a leukocyte differentiation antigen and a transmembrane receptor on T cells. CTLA-4 shares CD28 with the B7 molecular ligand, which inhibits CTL activation by preventing the binding of the B7 ligand to CD28 (50). CTLA-4 is usually expressed in activated T cells to downregulate or terminate T cell activation and participates in negative immune regulation (80). CTLA-4 inhibitors have significant killing effects on solid tumors and are currently approved for of breast cancer treatment. However, their application in patients with EC remains in clinical trials (81). According to previous reports, the combined blockade of CTLA-4 and PD-1 amplifies anti-tumor T cell responses and provides synergistic activity (51). This combination therapy has been investigated in Phase III clinical trials (82). In 2018, the FDA approved the combination of ipilimumab and nivolumab for the treatment of MSI-H or MMRd metastatic colorectal cancer based on the CHECKMATE 142 study. This combination therapy has also been studied for gynecological cancer and has shown good clinical efficacy in patients with EC (83). Taylor et al. (84) reported that the efficacy rate of this combination therapy could reach 50%.

##### 2.4.1.3 B7-H3

B7-H3 is often overexpressed in tumors. This is related to tumor immune escape. As is shown in Brunner’s study, B7-H3 is expressed in most EC, and its high expression is closely associated with high-risk tumors. Therefore, EC B7-H3 is regarded as a negative regulatory factor for T-cell activation and may protect tumor cells from immune system surveillance (52). In addition, the expression of B7−H3 in EC cells positively correlated with the frequency of CD8^+^ tumor-infiltrating lymphocytes (TILs) and the overexpression of B7−H3 in EC cells was associated with short OS time (52).

##### 2.4.1.4 B7-H4

B7-H4, discovered in 2003, is a co-inhibitory molecule that negatively modulates T cell immune responses and promotes immune evasion by inhibiting T lymphocyte proliferation and cytokine secretion (53). However, increasing evidence suggests that B7-H4 in tumor cells is related to the inhibitory microenvironment of T lymphocytes and can limit tumor growth in animal models (85). Similar to the results of this study, Rahbar et al. (86) found that high expression of B7-H4 protein in breast cancer tissues was associated with a favorable prognosis for patients. Miyatake et al. (54) reported that B7−H4 was upregulated in malignant EC and was more common in advanced subgroups. Additionally, Bregar et al. suggested that the expression of B7−H4 was an independent factor of EC grade, histological type, and infiltrating−immune cell type (55).

#### 2.4.2 HLA

Because various tumor cells lack the expression of HLA class I molecules, they are unable to present immunogenic peptides to CTL that cannot be activated, leading to the immune escape of tumors. Researchers studied 486 patients with sporadic EC and found that HLA class I molecules were downregulated on the surface of the EC (56). Compared with patients with normal HLA expression, these patients had a significantly decreased number of CD8+ CTL in the tumor microenvironment. These patients have decreased disease-free survival rates. This suggests that EC escape the immune system by downregulating the expression of HLA class I molecules in the cell membrane (57, 87, 88). A previous study which involving 486 EC patients showed that the loss of HLA was 41.3% and that HLA was associated with high grade of EC (57). Furthermore Ben et al. (58) reported that the HLA level in patients with early stages of EC was high, which is consistent with a previous study.

#### 2.4.3 LAG-3

LAG-3, also known to as CD223, is primarily expressed in activated T and NK cells. LAG-3 can inhibit immune cell proliferation and cytokine release when activated by the major histocompatibility complex II (MHC II) (59). Studies have reported that binding to its ligand LAG-3 can induce the suppression of T-cell and thus causing tumor evasion (89). Preclinical studies have shown that various solid tumors, including colorectal, ovarian, and renal cancers, are associated with LAG-3, suggesting that LAG-3 is a promising immune checkpoint for the development of immunotherapies. Therefore, several clinical trials of ICIs targeting LAG-3 are currently associated with advanced solid tumors [NCT03743766, NCT02519322, NCT03662659, NCT03610711, NCT03459222, NCT03499899] (90). Recently, Zhang et al. (60) conducted a retrospective study of 421 patients with EC and found that LAG-3 expression in immune cells was more common in high-grade, high-intermediate risk, high-risk, and advanced/metastatic subgroups and was relevant to lymphovascular space invasion. Furthermore, LAG-3 expression was more prevalent in POLEmut and MMRd EC than in p53abn and p53wt EC in tumor cells (34.4% and 66.3% in POLEmut and MMRd versus 28.6% and 19.5% in p53abn and p53wt, P < 0.001). The positive expression of LAG-3 in tumor cells is associated with high levels of CD8+ T cell immune infiltration. In addition, positive LAG-3 expression may be a predictor of improved RFS (60).

Other novel immune checkpoint molecules that are not yet in clinical use include moiety 2,3-dioxygenase inhibitors (IDO), and T-cell immunoglobulin and mucin domain-containing protein 3 (TIM-3), and T-cell immunoglobulin and ITIM domain protein (TIGIT).

### 2.5 Inflammatory factors

Inflammatory cells can release IL-6 and TNF-α cytokines to affect cell proliferation signals. Cytokines can promote rapid cell proliferation and differentiation and increase the probability of abnormal mutations. For example, TNF-α at high concentrations can kill EC tumor cells. Meanwhile, EC tumor cells can release TNF-α causing DNA damage and abnormal cell repair (91). TNF-α can enhance tumor invasion by affecting angiogenic factors. Inflammatory cells can also promote the production of cyclooxygenase 2 (COX-2), which participates in tumor generation and invasion by affecting IL-6 and IL-11. Furthermore, inflammatory cells can produce local active nitrogen clusters and reactive oxygen clusters, which can cause cell DNA damage, and easily form abnormal mutations that can promote growth and tumor invasion (92). Che et al. (93) suggested that IL-6 promotes autosynthesis and EC growth via autocrine feedback mediated by the ERK-NF-κB signaling pathway. High expression rates of colony stimulation factor-1 (CSF-1), TNF-α and IL-6 were associated with poor prognosis in EC patients. Lay et al. (92) suggested that IL-11 promotes EC progression by activating STAT. These cytokines may be involved in the immune escape in EC.

### 2.6 ITME in EC revealed by single-cell RNA sequencing

ScRNA-seq is a method to measure the expression levels of all genes from individual cells, and reveals heterogeneity at cell level (94, 95). Liu et al. (96–99) applied ScRNA-seq to tumor tissues from patients of cervical cancer to explore the ITME. With the aid of scRNA-seq, the understanding of the ITME in gynecological tumors is improved. Several studies have been studied on EC using scRNA seq. Huang et al. (100) revealed that the small GTPase 3 (RAC3) was specifically distributed in EC tumor cells compared to normal tissues. High levels of RAC3 in EC tissues were reversely associated with CD8^+^ T cell infiltration. Furthermore, RAC3 accelerated tumor cell proliferation and inhibited its apoptosis, without impacting cell cycle stages. Importantly, silencing RAC3 improved the sensitivity of EC cells to chemotherapeutic drugs (100). Yu et al. (101) suggested EC cells can confer malignant phenotype to endothelial cells by Midkine (MDK)-nucleolin (NCL) signal and NCL is associated with suppressed immune activity. EC cells may shape ITME by inhibiting immune cells via MDK-NCL signal. Guo et al. (102) suggested the percentage composition of monocytes, DC and mast cells were higher in paratumor than in tumor. On the other hand, the percentage composition of macrophages was higher in tumor than in paratumor. They found that tumor infiltrating macrophages were associated with increased OS. Wu et al. (103) identified three macrophage subsets, and two of them showed tissue-specific distribution. The tumor-enriched macrophage subset was found to predict immunotherapy responses in EC. Furthermore, six genes were selected from macrophage subset markers that could predict the survival of EC patients, SCL8A1, TXN, ANXA5, CST3, CD74 and NANS, and a prognostic signature was constructed. Further research is needed to study ITME through scRNA-seq.

### 2.7 ITME in different subtypes of EC

To date, studies on ITME in EC have mostly focused on POLE mutations and the MMRd subtype. According to the TCGA data, patients with POLE mutations showed the highest tumor mutation burden (TMB), followed by those with the MSI-H subtype. Reports have illuminated that TMB is highly associated with tumor-infiltrating immune cells, PD-L1 expression and patients’ prognosis in EC (104–106). Meanwhile, studies have also reported that POLEmut and MMRd EC have a high number of CD8+ CTLs expressing PD-1 (29). This is the key mechanism of immune tolerance in POLEmut and MMRd EC, as PD-1 binds to PD-L1 to restricts CD8+ CTLs function. Furthermore, previous studies have shown that CD8^+^ CTLs generally indicate good prognosis (26). These results are consistent with each other, as in clinical practice, POLEmut and MMRd have relatively favorable prognoses and can benefit from immunotherapy. Fusco et al. (107) revealed that a possible hypothesis supporting these results is that tumors with more somatic mutations produce higher levels of immunogenic antigens. Consequently, these tumors can be detected by CD8+ CTLs and are less likely to progress to a late stage. Guo et al. (20) explored 123 MSI EC and found that MSI tumors were enriched with CD8+ CTLs, Treg cells, fewer M2 macrophages, activated DCs, and a higher trend of CD20^+^ B cells infiltration. Patients with MSI EC were identified more often in the early stages, had a lower age, and better survival. Julie et al. (44) found CTLA4, PD-1, PD-L1, TIM-3 and IL-6 expression significantly increased in all EC grades. The number of CD4^+^ T cells was similarly increased in all EC grades, whereas the number of CD8^+^ CTL was only increased in the grade 1 ECs and decreased or remained unchanged in others grades. Daniel (108) studied the POLEmut type and found similar results to those of the prominent CD8^+^ CTL present in POLE-mutant. Mohammad (109) performed a retrospective study and found that a combination of PD-L1 positivity and MMR deficiency may be associated with aggressive features such as LVSI. Dai et al. (7) conducted a retrospective study to comprehensively analyze the ITME of four molecular subtypes in EC comprehensively for the first time. They combined the POLE mutant and MSI-H subtypes into the TMB high (TMB-H) subtype owing to their small sample size. They analyzed the ITME features of 30 EC cases, including TMB, infiltration of anti-tumor-related immune cells and negatively regulatory immune cells, and expression of immune checkpoint molecules. Similar results were found in previous studies, which showed that POLE mutations showed the highest level of TMB, followed by the MSI-H subtype, NSMP, and TP53 mutant subtypes. The TMB-H subtype showed a high degree of infiltration of CD8^+^ T cells and relatively high levels of PD-L1 expression in tumor cells. Although TMB levels were low in the TP53 mutant subtype, the proportions of Treg, M2 macrophages, PD-L1^+^ CD68^+^ macrophages, and CD8^+^ PD-1^+^ T cells were relatively high, indicating a strong immunosuppressive microenvironment in this subtype. In the NSMP subtype, the TMB, proportions of multiple tumor-infiltrating immune cells, and expression levels of immune checkpoint molecules were low, indicating a lack of effective anti-tumor immune responses. Based on the immune microenvironmental features, they summarized the immune phenotype of the three molecular subtypes as normal immune response, absence of immune infiltration, and suppressed immune response. Due to the relatively small sample size, further studies with larger sample sizes are required to confirm these findings. The ITME in different subtypes of EC is shown in **Table 3**.

**Table 3:** Main Findings in The immunosuppressive tumor microenvironment of different EC subtypes.

Molecular subtype	Main findings
POLEmut	Have a high rate of CD8^+^CTLs with PD-1 (7, 26, 29). (104–106, 108) Have relatively favorable prognosis and can benefit from immunotherapy (26, 107).
MMRd/ MSI-H	Have a high rate of CD8^+^CTLs with PD-1 (7, 26, 29). (104–106) Have relatively favorable prognosis and can benefit from immunotherapy (26, 107). Enriched with CD8^+^ CTLs, Treg cells, fewer M2 macrophages, activated DCs, and a higher trend of CD20^+^ B cells infiltration (20). CTLA4, PD-1, PD-L1and IL-6 expression significantly increased in all EC grades. The number of CD4^+^ T cells was similarly increased in all EC grades, whereas the number of CD8^+^ CTL was only increased in the grade 1 ECs (44). a combination of PD-L1 positivity and MMR deficiency may be associated with aggressive features such as LVSI (109).
TP53abn	the proportions of Treg, M2 macrophages, PD-L1^+^ CD68^+^ macrophages, and CD8^+^ PD-1^+^ T cells were relatively high, indicating a strong immunosuppressive microenvironment in this subtype (7).
NSMP	proportions of multiple tumor-infiltrating immune cells, and expression levels of immune checkpoint molecules were low, indicating a lack of effective anti-tumor immune responses (7).

POLEmut, DNA polymerase epsilon ultramutated; MMRd, mismatch repair deficient; MSI-H, microsatellite instability hypermutated; p53abn, p53-abnormal; NSMP, no specific molecular profile; EC, endometrial cancer; PD−1, programmed cell death 1; PD−L1, programmed cell death 1 ligand 1; LVSI, lymphatic vascular space infiltration; CTLA-4, cytotoxic T-lymphocyte-associated protein 4.

### 2.8 Immunotherapy in EC

Immunotherapeutic strategies for EC are widely used and can be divided into three categories: anticancer vaccines, ICI, and immunomodulators. In recent years, with the development of tumor cells and the tumor immune microenvironment, immunotherapy, especially ICI, has been applied for the treatment of a variety of malignancies. Immune checkpoints regulate co-stimulation signaling to maintain immune self-tolerance in the body and prevent immune damage caused by the excessive activation of T cells. This is the mechanism by which tumor cells escape immune surveillance and death. By suppressing immune checkpoint activity, ICI activate the tumors recognition and killing functions of immune cells. At present PD-1/PD-L1 and CTLA-4 antibodies were widely used (110). These immunotherapies are most effective for POLEmut and MMRd tumors because of their high TMB and increased immunogenic antigens (111). The higher TMB and PD-L1 expression in MSI-H and MMRd endometrial tumors support the clinical efficacy of PD-1 inhibitors in treating solid tumors with MMRd (112). Moreover, as mentioned earlier, PD-L1 expression increased with TIL abundance in the EC ITME, indicating that PD-1 inhibitors can induce an effective anti-tumor immune response in EC with high PD-L1 expression (104–106). In 2017, the FDA approved pabolizumab for the treatment of solid tumors with unresectable or metastatic MSI-H or MMRd. In 2021, the FDA approved dostarlimab-gxly for adult patients with MMRd solid tumors (113). Furthermore the National Comprehensive Cancer Network (UCCN) and ESGO/ESTRO/ESP guidelines in 2020 recommended anti-PD-1 targeted therapy for patients with advanced MMRd EC (21). Multiple clinical trials are currently underway regarding the treatment of EC with PD-1 antibody. KEYNOTE-016 study, a phase II clinical study in the United States evaluated the efficacy and safety of pabolizumab in 41 cases of metastatic colorectal cancer. The objective response rate (ORR) of tumors with MMRd and proficient MMR (pMMR) were 40% and 0%, respectively (19). KEYNOTE-158 multicenter phase II clinical study further evaluated the efficacy and safety of pabolizumab in patients with MSI-H/MMRd endometrial cancer (114). GARNET Study, a phase I/IIb clinical study conducted in 117 centers in 9 countries, had the largest sample size of monotherapy with PD-1 antibody for advanced or recurrent EC. A total of 271 patients were enrolled in this subgroup, and dostarlimab (TSR-042) was administered to patients with MMRd and pMMR. The ORR for MMRd and pMMR patients were 44.7% and 13.4%, respectively (115). Based on the GARNET study, dostarlimab was approved by the FDA for monotherapy in adult patients with recurrent or advanced EC with MMRd after chemotherapy with previous platinum-containing drugs. ICI therapy can relieve the symptoms of some patients and improve their prognosis; however, many patients show primary or acquired resistance. Therefore, immunocombination therapy is necessary to improve the efficacy and mainly includes combined chemotherapy, immunotherapy, and targeted therapy. Several clinical studies have investigated the efficacy and safety of the combination of PD-1/PD-L1 and CTLA-4 antibodies in patients with advanced/recurrent EC (NCT03015129, NCT03508570, and NCT02982486). With the expansion of clinical research on PD-1/PD-L1 antibodies, the management of their adverse reactions is particularly important. The anti-tumor mechanisms of PD-1/PD-L1 antibody and classical chemotherapy are different, so the adverse reaction profile is also quite different. The molecular mechanism is not yet clear and is mostly considered to affect immune homeostasis.

### 2.9 New subtypes of EC based on ITME—Immunophenotype

In 2020, Liu et al. (116) selected a series of immune-related genes between EC and normal endometria, and used Cox regression model analysis to select the genes related to prognosis, and obtained 15 immune-related genes to calculate the risk score. Patients were then divided into high and low-risk groups according to their risk scores. Li and Wen (73) also used immune-related genes in TCGA database to define four immune types: Immunosuppressive (type C1), Interferon γ-dominant (type C2), Inflammatory (type C3), and Immune balance (type C4) respectively. Bagaey et al. (117) performed tumor microenvironment typing based on the expression of immune-related genes in the ITME and identified four immune types: immune-enhanced fibrosis (type IE/F), immune-enhanced non-fibrosis (type IE), fibrosis (type F), and immune depletion (type D). Type E is the most effective immunotherapy and has the best prognosis. Thorsson et al. (118) used immune-related genes to obtain six immune-related types: tissue-based type (type 1), interferon γ-dominant (type 2), inflammatory (type 3), lymphocyte depletion (type 4), immune silent type (type 5), and transforming growth factor (type 6). Types 4 and 6 are mainly giant cells in the tumor microenvironment with less lymphocyte infiltration and the worst prognosis, whereas types 2 and 3 have the best prognosis.

Some investigators have performed immunotyping based on the infiltration of immune cells into the tumor tissue. Cai et al. (119) clustered samples according to immune cell infiltration in tumor tissues and obtained three subsets, CI, CII, and CIII, whose immune cell infiltration was high, medium, and low, respectively. Most specific gene mutations were detected in CI and CII, whereas a higher frequency of TP53 gene mutation and copy number variations were found in CIII. Wang et al. (120) combined the degree of immune cell infiltration with gene expression levels to calculate the tumor microenvironment score and divided EC into high- and low-risk groups. Immune activation and immune checkpoint related genes were frequently expressed in low-risk groups. The high-risk group had a higher frequency of PTEN, CSE 1 L, ITGB3 mutations. These studies indicate that immune features in the EC tumor microenvironment are related to prognosis and can be used as a new basis for EC classification.

## 3 Conclusion

EC is an immune gene disease. Patients with early stage of EC have a favorable prognosis, whereas those with advanced EC have a worse prognosis and respond poorly to treatment. Therefore, immunotherapy has attracted attention, and researchers have focused on the ITME of EC in recent years. Since 2014, the FDA has approved several ICIs in clinical practice, mainly for patients with advanced or recurrent cancer who have failed conventional treatment. However, the relatively low response rate limits their application in clinical practice, and the efficacy of immunotherapy is not satisfactory (121). Therefore, screening the dominant therapeutic population when using ICIs is crucial. The classification method for EC has been continuously improved, gradually changing from traditional clinical and pathological classification to molecular classification. Research on the microenvironment of EC based on molecular typing remains unclear. Most studies have focused on POLEmut and MMRd types, and their results were consistent in that the two types have a high rate of CD8^+^ CTLs with PD-1, a relatively favorable prognosis, and can benefit from immunotherapy. Dai et al. (7) comprehensively studied the ITME of four different EC molecular subtypes for the first time and found that the TP53 mutant subtype had a relatively high proportions of Treg cells, M2 macrophages, PD-L1^+^ CD68^+^ macrophages, and CD8^+^ PD-1^+^ T cells, indicating that the TP53 mutant subtype could benefit from immunotherapy to some extent. Furthermore, in the NSMP subtype, which accounted for 31.5% according to the data published by TransPORTEC-3, the TMB, the proportions of multiple tumor-infiltrating immune cells and the expression levels of immune checkpoint molecules were low. It is important to investigate whether these patients could benefit from immunotherapy, as the P53abn and NSMP subtypes were enriched with immune cells to some extent. This suggests that the P53abn and NSMP subtypes may also be suitable candidates for immune checkpoint-blocking therapy. However, few relevant studies on ITME have been reported, and most are retrospective studies with small sample sizes. To clarify the microenvironment of various types of EC and identify biomarkers that can accurately predict the response to immunotherapy, prospective studies with large samples sizes are needed in the future.

With research on the immune microenvironment of EC, a new immunophenotype classification based on the immune microenvironment has been carried out in recent years. However, the immunophenotype of EC is still limited to the research stage and there are many limitations. First, owing to intratumor heterogeneity, the samples used for testing may hardly represent the overall immune environment of the tumor, resulting in inaccurate immunophenotyping. Second, most current studies have only analyzed the gene level and have not verified the actual infiltration of immune cells using methods such as immunohistochemistry. Third, some studies lack validation of independent cohorts and immunotyping models in clinical trials. Currently, molecular typing based on tumor cells is gradually being incorporated into clinical practice, providing a basis for the selection of adjuvant and immunotherapies for patients. However, immunotyping methods based on immune characteristics are still under study, and more clinical practice is needed to evaluate their benefits.

Therefore, further studies are required to promote the use of these immunotypes in clinical practice.

In conclusion, the advent of immunotherapy has brought hope for improved efficacy and prognosis in patients with advanced or recurrent EC. The efficacy and safety of ICIs combination therapy remains the focus of future research. In the future, additional potential molecular markers should be explored through molecular pathways and immune resistance mechanisms in the tumor microenvironment. In future research, the results may change the current limited treatment patterns, believing that more cancer patients will benefit from them.

## Author contributions

CZ: Writing – original draft, Writing – review & editing. MW: Supervision, Writing – review & editing. YW: Conceptualization, Writing – review & editing.
